# Chili cultivars vulnerability: a multi-factorial examination of disease and pest-induced yield decline across different growing microclimates and watering regimens

**DOI:** 10.1186/s12870-024-05541-3

**Published:** 2024-10-18

**Authors:** Farhan Ahmad, Kusumiyati Kusumiyati, Mochamad Arief Soleh, Muhammad Rabnawaz Khan, Ristina Siti Sundari

**Affiliations:** 1 Department of Agronomy, Agricultural Faculty, Universitas Padjadjaran, Jl. Bandung-Sumedang km 21 Jatinangor, Sumedang, West Java Indonesia; 2 Department of Agronomy, Faculty of Crop Production Sciences, The University of Agriculture Peshawar, Peshawar, Khyber Pakhtunkhwa Pakistan; 3 Department of Agribusiness, Faculty of Agriculture, Universitas Perjuangan, Jl. PETA No. 177 Tasikmalaya, Tasikmalaya, West Java Indonesia

**Keywords:** Crop resilience, Plant physiology, Environmental stressors, Microclimate, Yield optimization

## Abstract

**Background:**

As identified by the research, it is imperative to develop effective ways to address the pressing problem of disease and pest susceptibility in chili agriculture and secure sustainable crop yield. The research examines the impact of various growing microclimates, watering regimens, and chili cultivars on disease incidence, pest attacks, and yield loss.

**Results:**

The study, which took place over a season, used a randomized complete block design to evaluate how well Tanjung, Unpad, and Osaka cultivars performed in four different watering regimens (100, 75, 50, and 25% ETc) and different microclimates (greenhouse, rain shelter, screen house, and open field). The findings exhibited that watering regimens and microclimates greatly influenced disease and pest occurrence, but cultivars had a minimal effect on these variables. Disease and pest attack rates were highest in the open field and lowest in the screen house. A correlation was found between lower disease and pest incidence and optimal irrigation levels (75% and 100% ETc). At lower watering regimens of 25% ETc and in the open field, yield loss was the greatest.

**Conclusion:**

The results emphasize how crucial controlled environments and appropriate irrigation techniques are to reducing crop loss and increasing production. Enhancing watering regimens and implementing screen house cultivation are two strategies for improving the productivity and sustainability of chili output.

## Introduction

Chili pepper (*Capsicum* *annuum* L.) is a widely consumed vegetable in Indonesia that enhances a variety of foods with its fiery taste. Growing chili peppers brings multiple challenges, such as fungal diseases, viral infections, and insect infestation, which can drastically lower crop productivity [1]. Climate change dramatically impacts chili, which causes shifting agroecosystem borders, invading pests, and lower crop yields. Elevated temperatures related to climate change may boost pest numbers and metabolic rates, further harming crops [2]. On average, chili yield reductions of 4% were typical signs of anthracnose disease. However, 22% of fungal diseases and 25% could decline as diseases spread [3]**.** It has been demonstrated that anthracnose infestations reduce chili crop yields by 10–80% during the rainy season and 2–35% during the dry season. Epidemics of anthracnose not only result in significant losses to the harvest but also worse quality products [4]**.**

Microclimate offers distinct characteristics, affecting plant health, pest pressure, and overall productivity [5]. Plant development is optimized by the enclosed growing, which offers an ideal microclimate with regulated levels of light, humidity, and temperature. Greenhouses provide an environment favourable to some pests and diseases, including powdery mildew, aphids, and whiteflies [6]. Because of their high humidity, uniform temperatures, and lack of natural predators, greenhouses foster an environment favourable to diseases and pests [7]. Greenhouses' confined airflow and elevated humidity levels might reduce crop yields if fungal diseases and pest infestations are not adequately controlled. Rain shelters minimize the incidence of fungal infections and fruit rot by protecting plants from direct exposure to rainfall [8]. It reduces the yield losses that are associated with these pathogens [9]. Screen houses provide the proper balance of ventilation to promote healthy plant growth while protecting from diseases and pests. Pest pressure, such as aphids, thrips, and leaf miners, which can seriously damage chili crops, is substantially reduced by screen house growth [10]. Open fields provide ample space for crop growth and exposure to natural light, but they also subject plants to various environmental stresses, such as strong winds, flooding, and pests [11].

Ensuring crop health, nutrient uptake, and soil moisture levels all depend on implementing suitable watering regimes. Inefficient watering techniques make chili plants more susceptible to pests and diseases, which lowers their production [1]. Higher yield results from the adequate regimen's promotion of healthy root development, nutrient uptake, and fruit set through the maintenance of proper soil moisture [12]. The crop experiences severe water stress due to a limited watering strategy, which compromises plant resistance and output [13]. Regulation of stomata and osmotic adjustment are adaptive ways that chili plants can respond when there are less water available, extended drought circumstances may compromise crop survival and yield potential [14].

The performance and productivity of chili crops are greatly influenced by cultivar selection, which also affects traits including fruit quality, disease resistance, insect tolerance, and prospective yield [15]. Determining whether cultivars are more vulnerable to pests and diseases is crucial for the sustainable production of chilies, as these issues are becoming more and more of a problem in the field [16]. Differential responses to biotic stressors from genetic trait variability among cultivars might impact overall production and quality [17]. Determining which chili cultivars are susceptible to disease and pest-induced yield decline is essential for guiding cultivar selection and management strategies in systems that produce chilies [18].

This study investigates how different cultivars of chilies react to yield declines caused by pests and diseases in various microclimates and watering regimens. By examining the various aspects influencing chili production, the research aims to offer important information for maximizing cultivar selection and management strategies, ensuring the sustainable cultivation of chili peppers.

## Materials and methods

### Experimental site and materials

This experiment was conducted at the Bale Tatanen Greenhouse, and Field Laboratory of the Faculty of Agriculture, Padjadjaran University in Jatinangor from December 2023 – May 2024.

The materials that used in the experimental setups containing chili cultivars (Local Tanjung, Unpad and imported Osaka) plastic container, laboratorium, knife, stick, mulch, scales, wrap, packaging, box, thermometer, hydrometer, thermohygrometer, stationary, label, scale glass, gallon 200 l, lux meter, anemometer.

### Treatment factors

**Table:** 

*Treatment factor 1Watering regimens (W)*	*Treatment factor 2 Growing microclimates (G)*	*Treatment factor 3* *Cultivars (C)*
*Level 1: 100% ETc (W1)*	*Level 1: Greenhouse (G1)*	*Level 1: Tanjung (C1)*
*Level 2: 75% ETc (W2)*	*Level 2: Rain shelter (G2)*	*Level 2: Unpad (C2)*
*Level 3: 50% ETc (W3)*	*Level 3: Screen house (G3)*	*Level 3: Osaka (C3)*
*Level 4: 25% ETc (W4)*	*Level 4: Open field (G4)*	

### Plant material

The seeds of chili cultivar Tanjung, Unpad and Osaka were prepared on germination pot trays. The germination medium was the mixture of compost and cocopeat. The pot tray was arranged as per the treatments of experiment design. Pot tray germination was stored in germination room in greenhouse. Once the seed germinate 3 or four foliar, it called seedling. The seedlings were planted using poly bags media in greenhouse, rain shelter, screen house and open field to grow as treatments of experiment design. The chili pepper plants were watered by four treatment watering regimens such as 100% FC, 75% FC, 50% FC and 25% FC. (The procedure for recording day to day watering was equipped in such a way that the three samples plant media were taken in each cultivar treatment and applied the water until field capacity and record the weight of the media and percolated water. Plant media was weighed on the following day as to note the difference of weight (evapotranspiration). The difference recorded was considered the evapotranspiration from that plant media and was assigned as 100% ETc. For 75% ETc the evapotranspiration of 100% was multiply by 0.75, for 50% multiply by 0.50 and for 25% by 0.25). All plants were in optimum maintenance. Harvesting time addresses to first, second, third, fourth and fifth or until the production reach lowest.

### Observations

1. Disease incidence rate (%)
  To record the disease incidence rate, determine the proportion of diseased chili plants by counting them and dividing the result by the total number of plants examined.
  $$Disease\;incidence\;rate\;\left(\%\right)=\frac{No.\;of\;diseased\;plants}{Total\;number\;of\;plants\;surveyed}x\;100$$
2. Pest severity (%)
  $$Pest\;severity\;\left(\%\right)\;=\frac{No.\;\;of\;pest\;affected\;plants}{Total\;number\;of\;plants\;surveyed}x\;100$$
3. Disease progression (%)
  Tracking the evolution by consistently observing and documenting the progression of disease symptoms.
  $$Disease\;progression=\frac{Final\;disease\;severity-Initial\;disease\;severity}{Time\;elapsed}$$
4. Disease index
  Following the scoring of individual plants, the following formula was used to determine the disease index:
  $$Disease\;index\;=\frac{Sum\;of\;severity\;ratings\;for\;all\;plants}{Plants\;observed}x\;100$$
  Severity rating (Table 1);

**Table 1:** Criteria for disease and pest severity rating (whole plant)

*Rating*	*Disease severity*
*1*	*5% plant affected*
*2*	*10% plant affected*
*3*	*15% plant affected*
*4*	*20% plant affected*
*5*	*25% plant affected*
*6*	*30% plant affected*
*7*	*35% plant affected*
*8*	*40% plant affected*
*9*	*45% plant affected*
*10*	*50% plant affected*
5. Disease nature
  The nature of the diseases was noted by visually inspecting (followed by google lens and expert) the fruit and foliage of the plants. It required carefully inspecting every plant for visible disease indicators, including wilting, leaf spots, necrosis, and other symptoms.
6. Disease & pest incidence stage
  Routine (daily at morning and evening) crop inspections were done for thorough monitoring to record disease and pest symptoms and severity at the vegetative, flowering, fruiting, and maturity stages.
7. Fruits affected by pest & diseases
  Examine every fruit for signs of pests or diseases, keeping note of any abnormalities, and report results in a systematic manner.
8. Recovery rate (%)
  The percentage of plants that have recovered about the total number of plants infected can be observed by this calculation. The method may assist in monitoring the success of efforts to recover and evaluate the growth of plant health management strategies.

$$Recovery\;rate=\frac{No.\;of\;recoverd\;plants}{No.\;of\;affected\;plants}x\;100$$

### Data analysis

The experiment design using Factorial Randomized Complete Block Design. The main benefit of implementing a Randomized Complete Block Design (RCBD) factorial over a split-plot design is minimizing block variations, thus providing a more accurate and efficient assessment of treatment effects and interactions. It improves the experiment's precision and enables more effective control over confounding variables. The constructed mathematical linear model equation as following:

$${\mathbf Y}_{\mathbf{ijk}}\boldsymbol=\mathbf u\boldsymbol+{\mathbf W}_{\mathbf i}\boldsymbol+{\mathbf C}_{\mathbf j}\boldsymbol+{\mathbf P}_{\mathbf k}\boldsymbol+{\mathbf{\left(\mathrm{WC}\right)}}_{\mathbf{ij}}\boldsymbol+{\mathbf{\left(\mathrm{WP}\right)}}_{\mathbf{ij}}\boldsymbol+{\mathbf{\left(\mathrm{CP}\right)}}_{\mathbf{ij}}{\mathbf{\left(\mathrm{WCP}\right)}}_{\mathbf{ij}}\boldsymbol+{\mathbf r}_{\mathbf l}\boldsymbol+{\mathbf\varepsilon}_{\mathbf{ijk}}$$

If there is an interaction effect, then hypothesis testing regarding the main effect does not need to be carried out. Testing the main effect is useful if the interaction effect is not significant. Post-hoc test is done to find out the best treatment partially by Tukey Multiple range comparison test.

The Procedure of Tukey (HSD) is:

- Order treatments mean accordingly
- Formula uses as follow:
  $${\varvec{\omega}}={{\varvec{q}}}_{\propto }({\varvec{p}},{\varvec{v}})\sqrt{\frac{{\varvec{S}}}{{\varvec{r}}}}$$
  Where: *p* = treatment amount = t, v = error degree of freedom, r = replication amount, α = confident level, q_α_(p, v) = critical value that obtained from t-student table.
- Test criteria
  Compare absolute value of two different means that were distinguish the differences to HSD score.
  If |µ_i_ - µ_j_| > HSD_005_, means the test result is significant
  If |µi - µj| < HSD005, means the test result is not significant

### Climate of the experimental sites

**Fig. 1:**
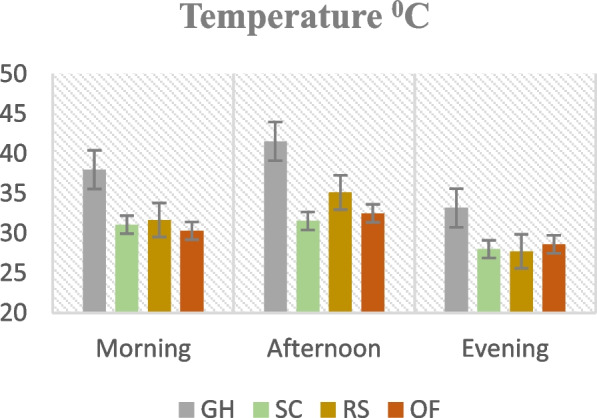
Mean temperature, humidity, wind velocity, and light intensity in the experimental sites from the month November till March. GH-Greenhouse, SC-Screen house, RS-Rain shelter, OF-Open field

**Fig. 2:**
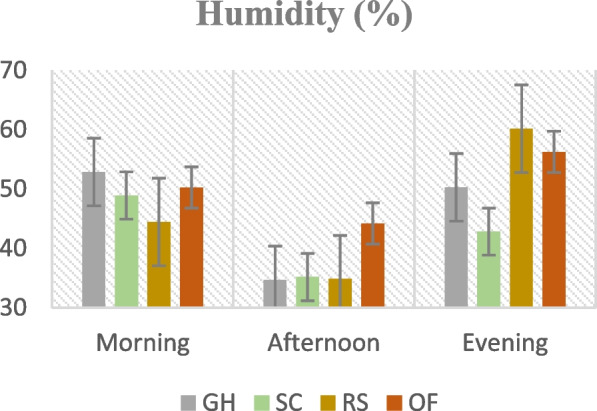
Mean temperature, humidity, wind velocity, and light intensity in the experimental sites from the month November till March. GH-Greenhouse, SC-Screen house, RS-Rain shelter, OF-Open field

**Fig. 3:**
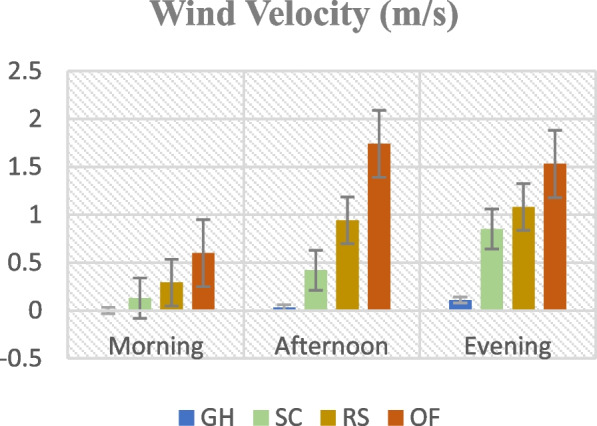
Mean temperature, humidity, wind velocity, and light intensity in the experimental sites from the month November till March. GH-Greenhouse, SC-Screen house, RS-Rain shelter, OF-Open field

**Fig. 4:**
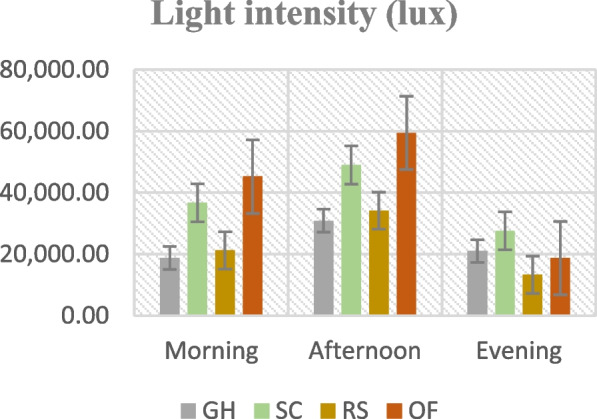
Mean temperature, humidity, wind velocity, and light intensity in the experimental sites from the month November till March. GH-Greenhouse, SC-Screen house, RS-Rain shelter, OF-Open field

Figures 1, 2, 3 and 4. Mean temperature, humidity, wind velocity, and light intensity in the experimental sites from the month November till March.

The devices used for recording climatic observations includes; Thermometer for temperature fixed in each growing microclimate, Hygrometer for humidity, Lutron LM-8000A digital anemometer for wind speed, Lux meter MT-30 for light intensity.



## Results and discussion

### Disease incidence %

**Fig. 5:**
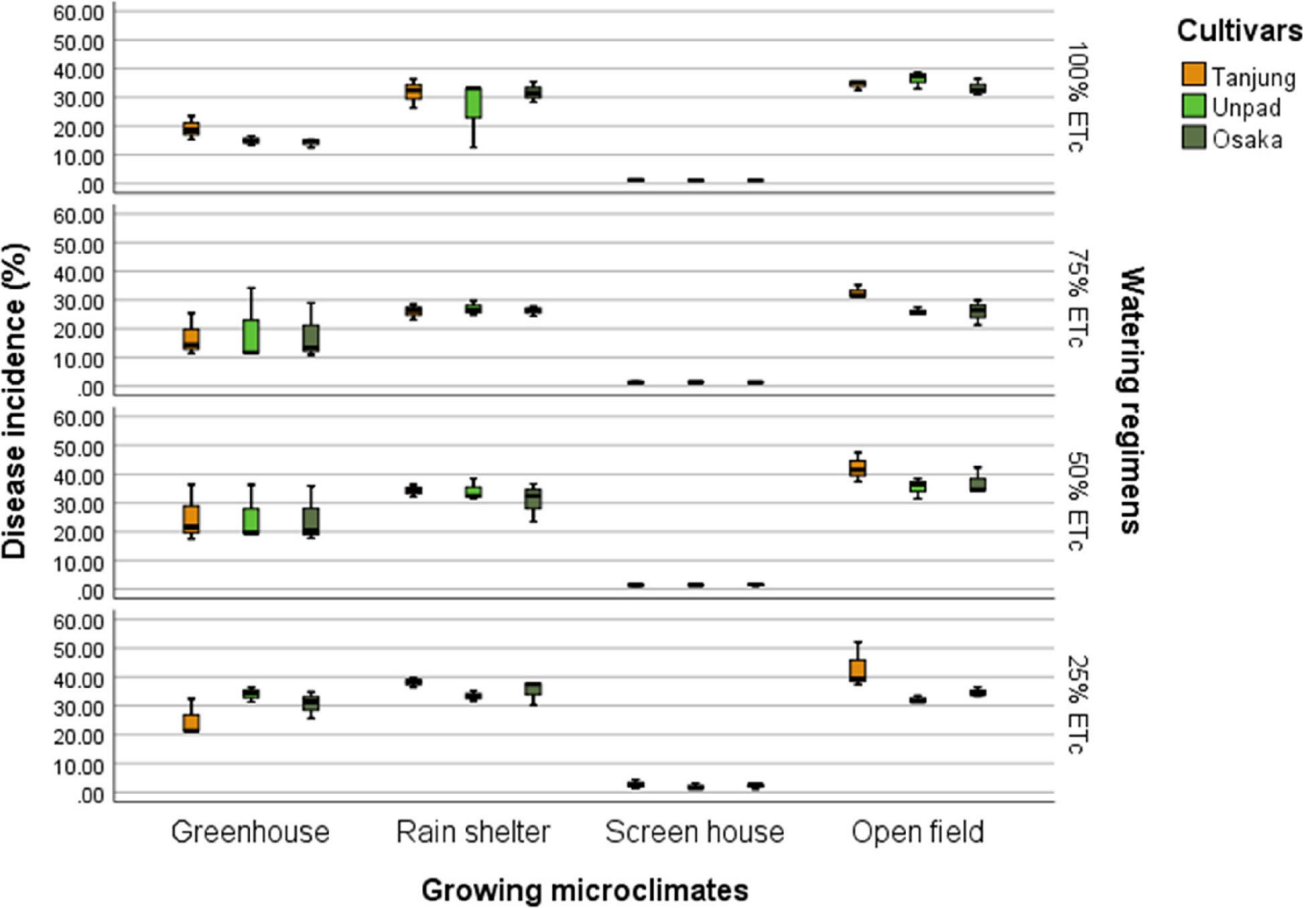
Mean disease incidence (%) of chili pepper cultivars as affected by different growing microclimates and watering regimens

Cultivars had a statistically insignificant effect on disease incidence, in contrast to the significant (*P* < 0.05) impacts observed in growing microclimates and watering regimens (Tables 2 & 3). Regarding disease incidence across the various microclimates, the open field had the highest score of 34.43%, followed by the rain shelter at 31.25%. The greenhouse had a lower score of 22.24%. In comparison, the screen house was the lowest at 1.52%. Regarding watering regimens, the maximum disease incidence was observed at 25% and 50% levels, at 26.08% and 24.45%, respectively. In comparison, the lowest incidence rates were observed at 100% ETc and 75% ETc levels, at 20.45% and 18.46%, respectively (Fig. 5).

**Table 2:** Mean values of the recorded observations, showing the significant differences (*P* < 0.05) among the treatment by lettering (x^a, b & c^) respectively

Cultivars	Disease incidence	Pest attack %	Disease progression %	Yield loss %	Disease index	Fruit affected by disease	Fruits affected by pest	Recovery rate
Tanjung	23.45^a^	30.72^a^	0.113^a^	21.52^a^	1017^b^	12.02^a^	11.48^b^	64.85^b^
Unpad	21.87^a^	30.15^a^	0.109^a^	20.47^b^	1048^a^	11.75^ab^	11.85^b^	65.82^b^
Osaka	21.76^a^	29.43^a^	0.114^a^	20.94^ab^	1039^ab^	10.38^b^	13.08^a^	69.32^a^
Growing designs								
Greenhouse	22.24^c^	38.06^b^	0.145^a^	20.24^c^	1147^b^	10.50^c^	12.56^b^	70.43^b^
Rain shelter	31.25^b^	39.04^ab^	0.122^b^	29.39^b^	1388^a^	18.39^a^	15.64^a^	51.23^d^
Screen house	1.52^d^	2.63^c^	0.037^c^	1.15^d^	202^c^	2.67^d^	3.36^c^	86.12^a^
Open field	34.43^a^	40.66^a^	0.143^a^	33.14^a^	1403^a^	15.03^b^	16.80^a^	53.58^c^
Watering volumes								
100% ETc	20.45^b^	28.8^b^	0.101^b^	19.37^c^	889^c^	9.86^b^	10.61^c^	71.83^a^
75% ETc	18.46^b^	29.05^b^	0.100^b^	19.18^c^	1029^b^	10.08^b^	11.53^bc^	66.80^b^
50% ETc	24.45^a^	30.32^ab^	0.111^b^	21.30^b^	1061^b^	12.64^a^	12.17^b^	62.25^c^
25% ETc	26.08^a^	32.23^a^	0.136^a^	24.06^a^	1160^a^	14^a^	14.25^a^	60.48^d^

The lettering (^a, ab, b, c^) showing the significant differences (*P* < 0.05) between treatments based critical value for comparison (LSD values)

**Table 3:**
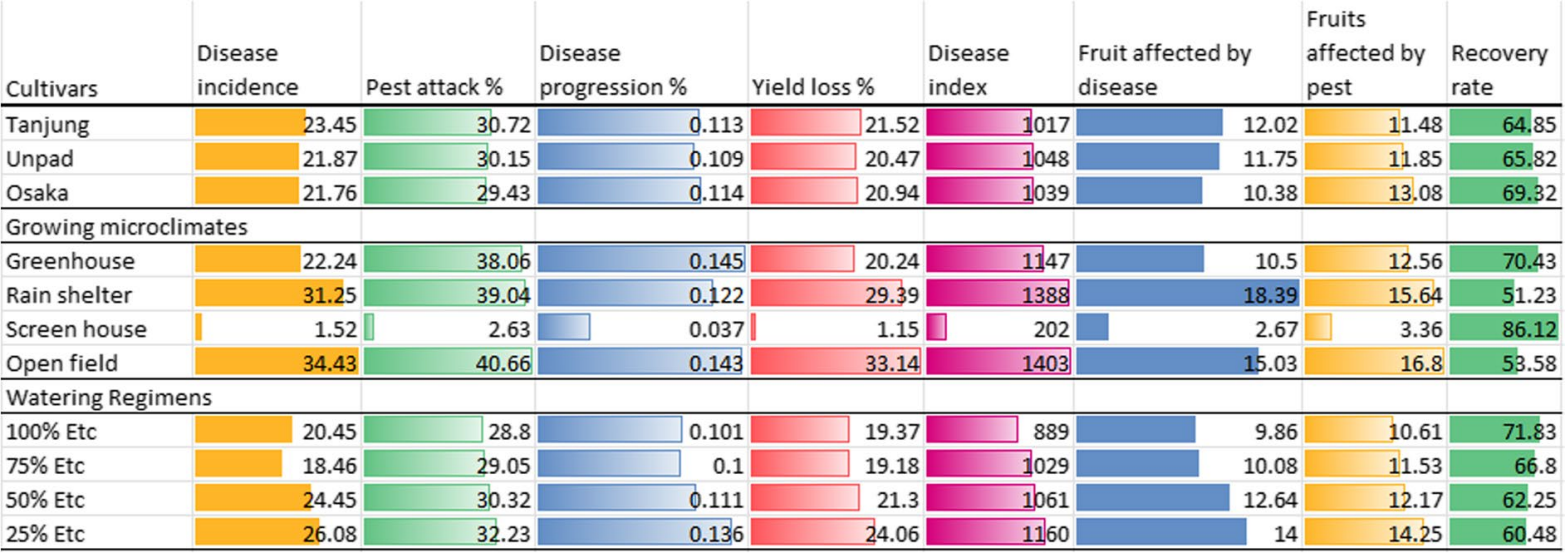
Showing the major variations of the data among the treatment by heatmap terminology

The heatmap demonstrates that the best practices for reducing disease incidence, insect assaults, and production loss in chili cultivars are a screen house environment and a 100% ETc watering schedule. Among all the cultivars, Tanjung has the lowest disease index and the highest recovery rate. The worst disease progression, pest attacks, yield losses in the open field, and 25% ETc watering regimen indicate a less ideal growing microclimate for chili growth

### Pest attack %

**Fig. 6:**
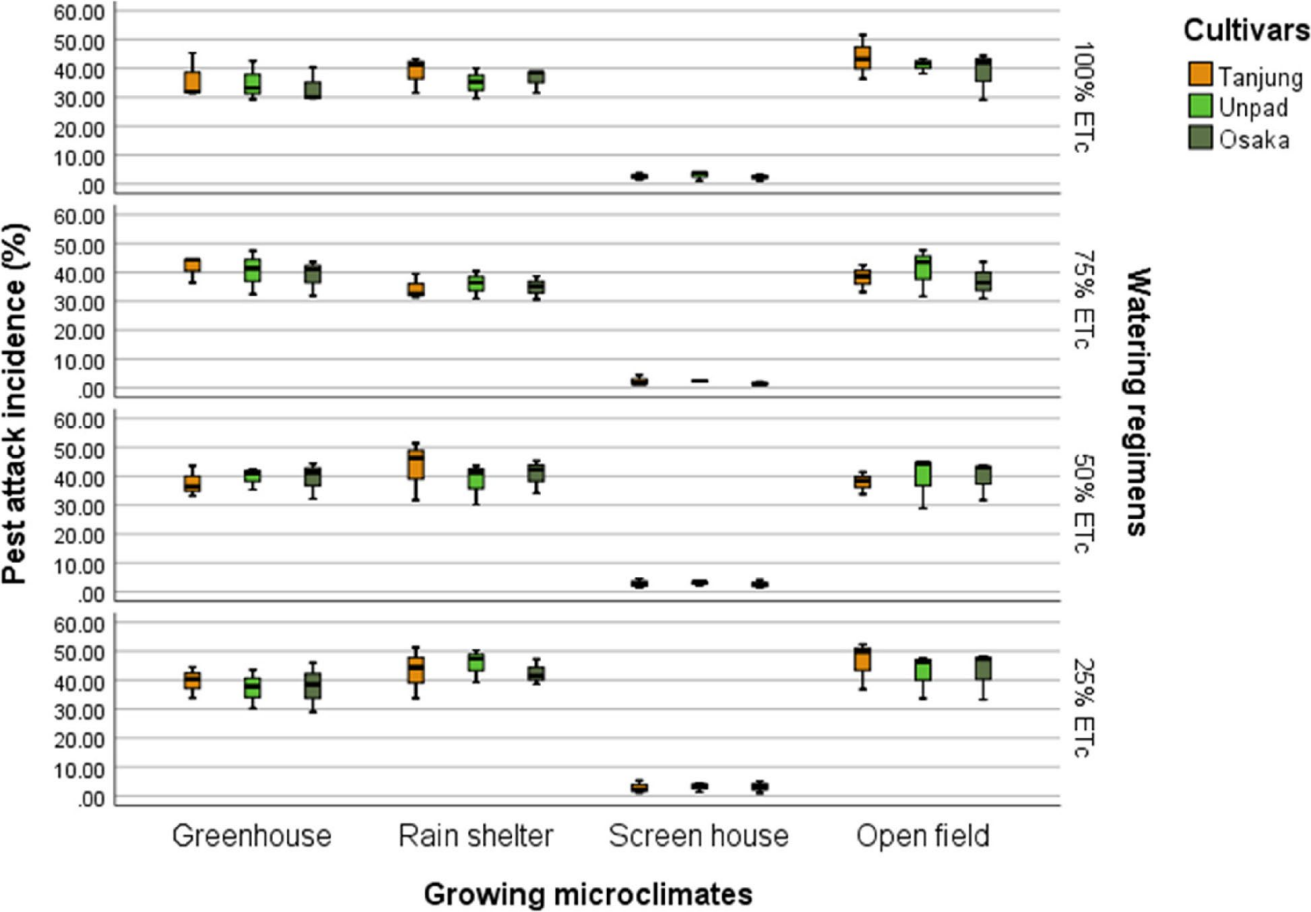
Mean pest attack incidence (%) of chili pepper cultivars as affected by different growing microclimates and watering regimens

Cultivars were found to have no significant (*P* > 0.05) effect on pest attacks in the research findings, in contrast to the significant (*P* < 0.05) impacts seen about growing microclimates and watering regimens (Tables 2 & 3). The open field had the highest percentage of pest attack (40.66%) among the different microclimates, followed by the greenhouse (38.06%) and rain shelter (39.04%). The screen house had the lowest rate of pest attacks (2.63%). Regarding the impact of watering regimens on pest attacks, the 25% ETc level was most affected (32.23%), followed by 50% ETc (30.32%), the 75% ETc affected lower (29.5%), whereas 100% ETc watering affected least (28.80%) (Fig. 6).

### Disease progression %

The research results showed that while cultivars had no significant (*P* > 0.05) effects on the progression of the disease, they did have significant (*P* < 0.05) effects on growth microclimates and watering regimens (Tables 2 & 3). The open field and greenhouse had the most significant disease spread rates among the microclimates under study, at 0.143% and 0.145%, respectively.

**Fig. 7:**
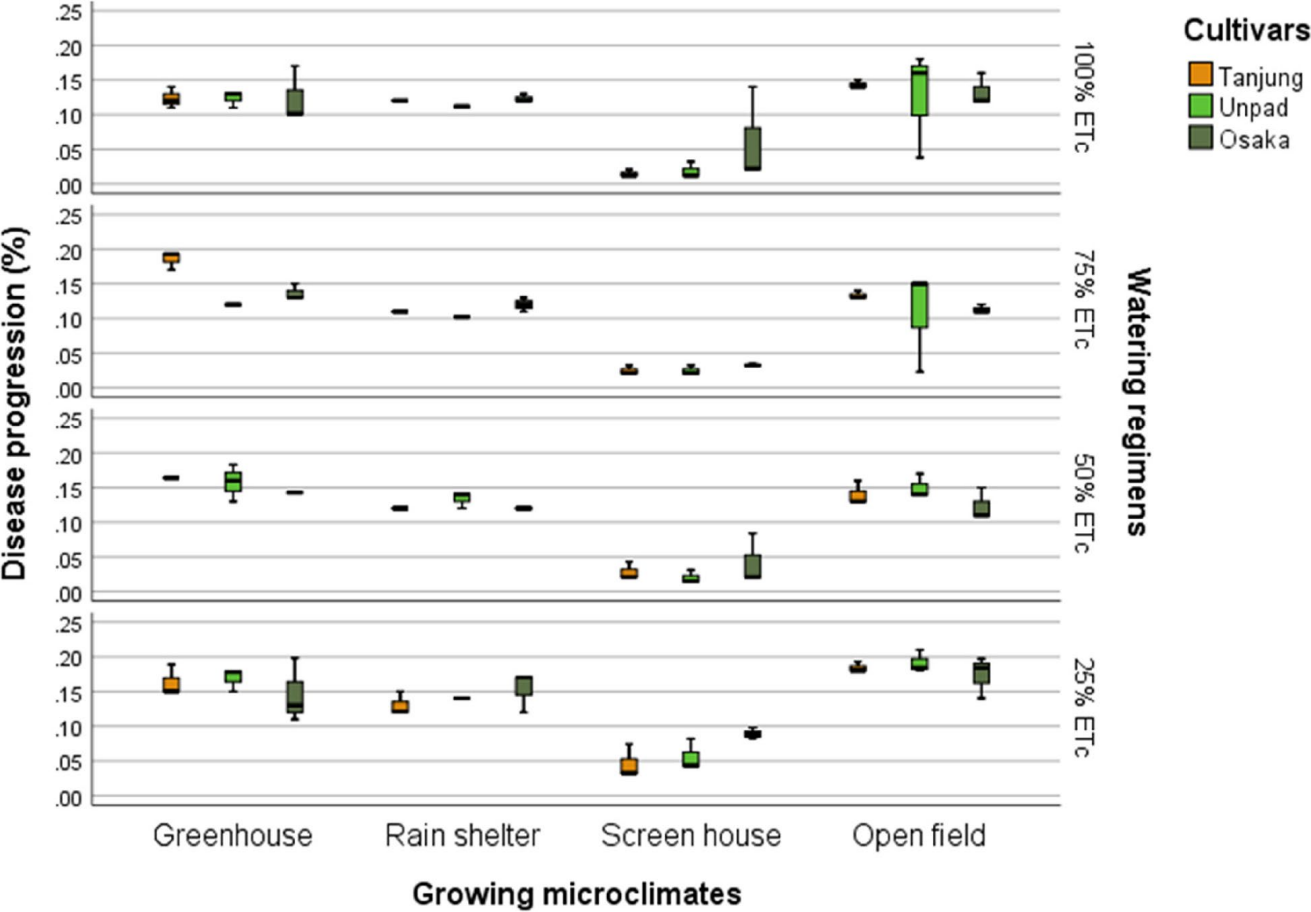
Mean disease progression (%) of chili pepper cultivars as affected by different growing microclimates and watering regimens

The rain shelter came in second, at 0.122%, while the screen house had the lowest rates, at 0.037%. In the analysis of how watering regimens affected the progression of the disease, the 25% ETc level revealed a greater rate of progression at 0.136%. In contrast, the greenhouse, rain shelter, and screen house rates were statistically similar at 0.101%, 0.100%, and 0.111%, respectively (Fig. 7).

### Yield loss %

**Fig. 8:**
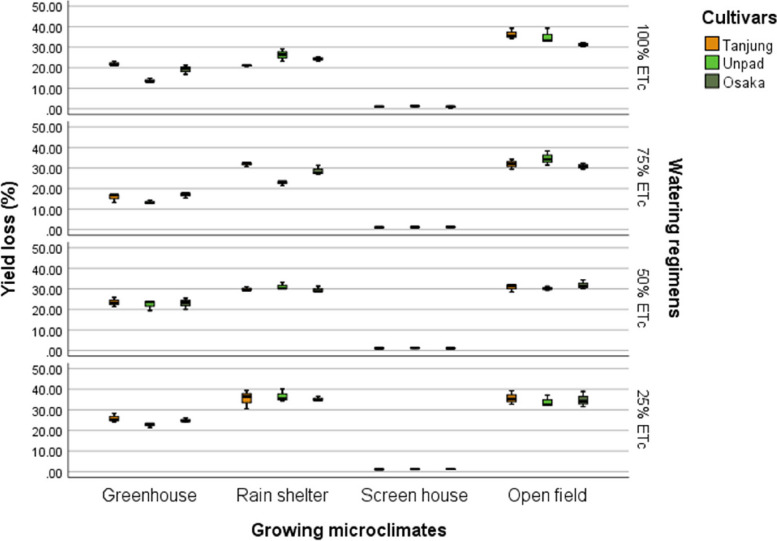
Mean yield loss (%) of chili pepper cultivars as affected by different growing microclimates and watering regimens

The research findings showed that cultivars, growing microclimates, and watering regimens significantly (*P* < 0.05) affected yield loss (Tables 2 & 3). Tanjung had the highest loss of any cultivar at 21.52%, followed by Osaka at 20.94% and Unpad at 20.47%. In terms of growing microclimates, the greenhouse showed a loss of 20.24%, the screen house the lowest at 1.15%, and the open field the most at 33.14%, followed by the rain shelter at 29.39%. Based on the analysis of watering regimens, the 25% ETc level had the most significant loss (24.06%), followed by the 50% ETc. The losses at the 75% and 100% ETc levels were statistically equal (19.18% and 19.37%, respectively) (Fig. 8).

### Disease index

**Fig. 9:**
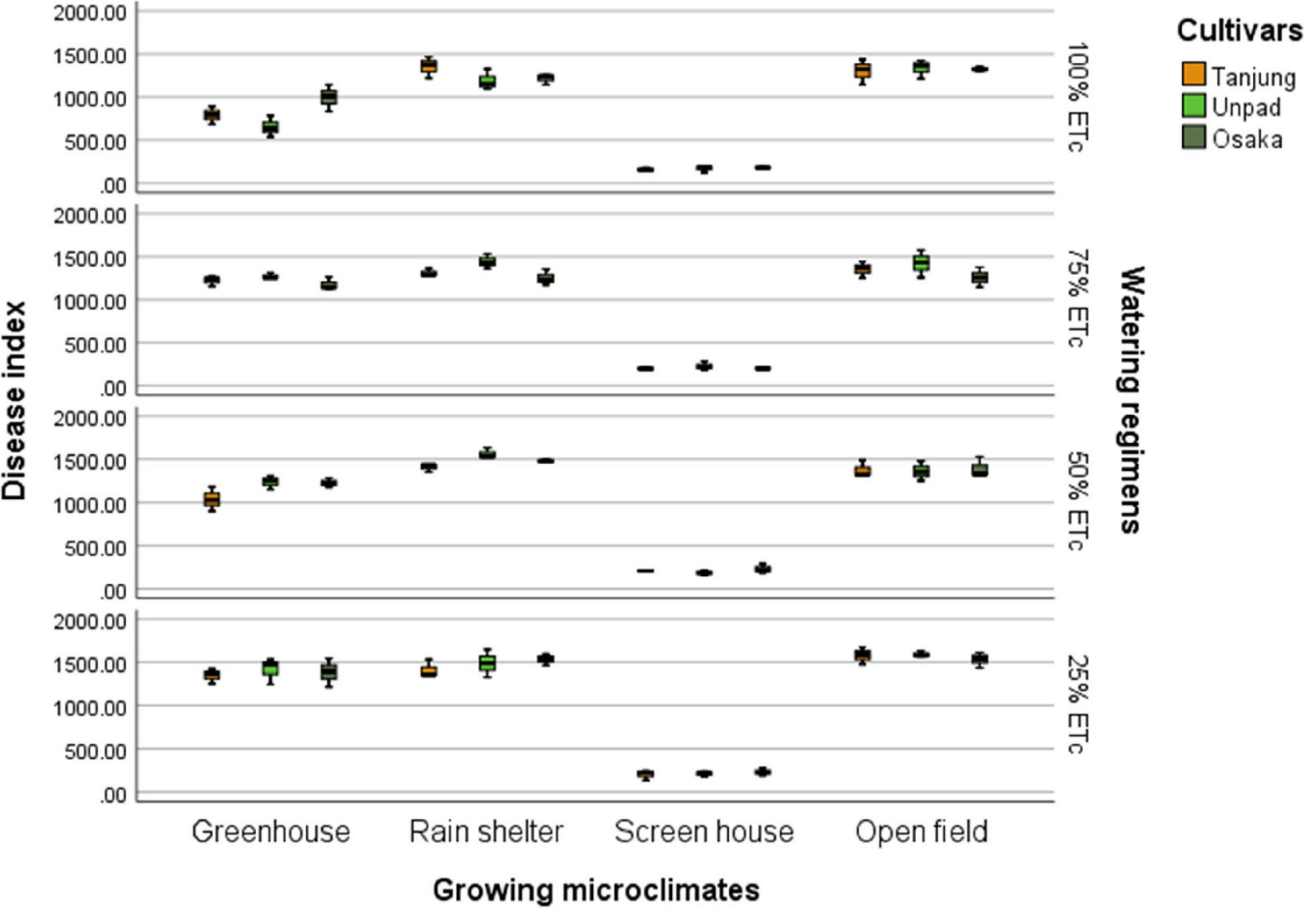
Mean disease index of chili pepper cultivars as affected by different growing microclimates and watering regimens

The results showed that watering schedules, growing microclimates, and cultivars all had significant (*P* < 0.05) effects on the disease index of chili (Tables 2 & 3). Tanjung had the lowest disease index (1017) among cultivars, while Unpad had the highest (1048), followed by Osaka (1039). The open field had the highest disease index for growing microclimates at 1403, followed by the rain shelter at 1388. The screen house showed the lowest disease index, at 202, while the greenhouse had the highest, at 1147. The 25% ETc level had the highest disease index (1160) regarding watering schedules, followed by the 50% ETc level (1061). The disease index for the 75% ETc level was 1029, while the lowest for the 100% ETc level was 889 (Fig. 9).

### Fruits affected by disease

**Fig. 10:**
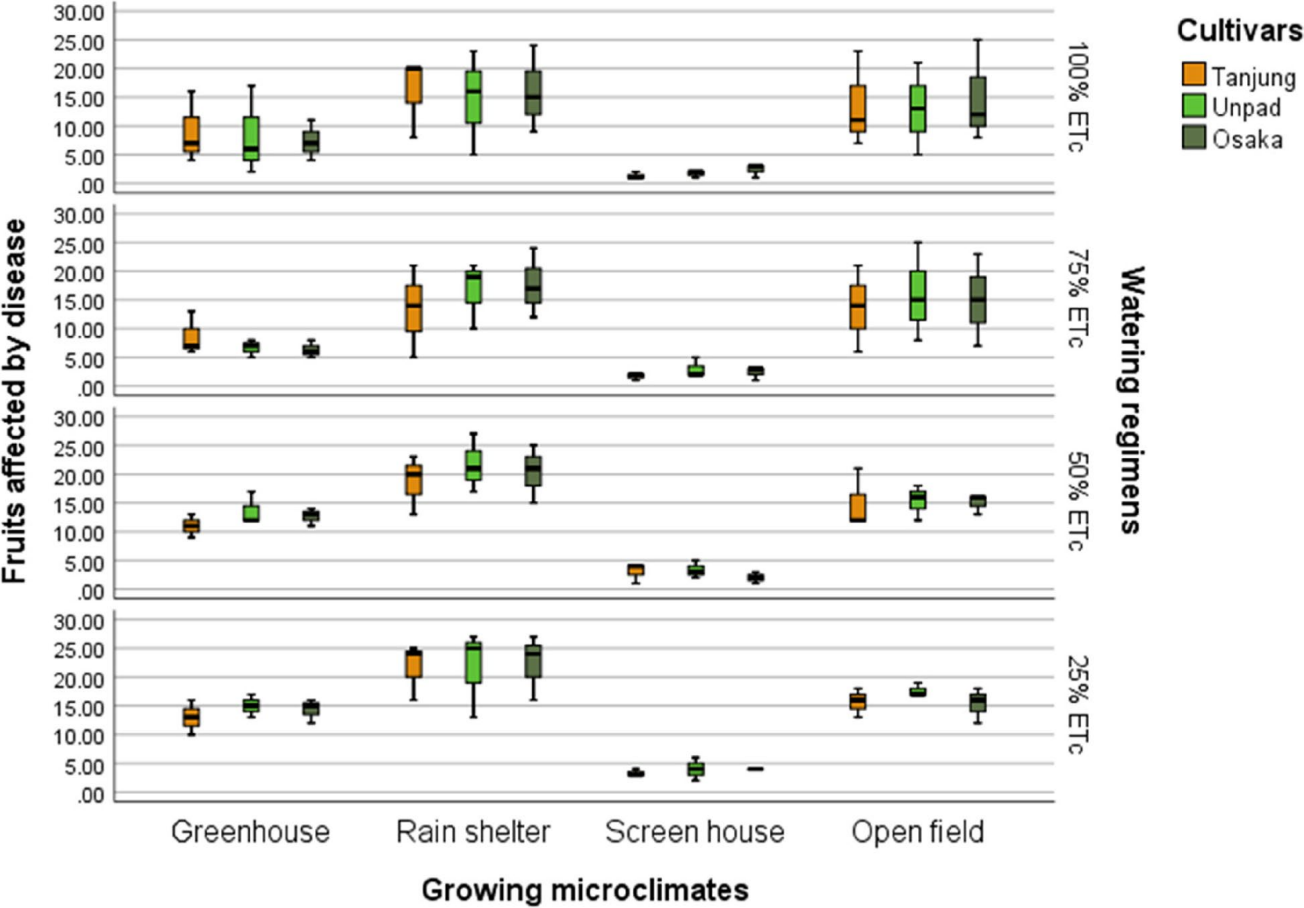
Mean fruits affected by disease of chili pepper cultivars as affected by different growing microclimates and watering regimens

The study's results revealed the significant (*P* < 0.05) impact of watering regimens, growing microclimates, and cultivars on the prevalence of disease in chili fruits (Tables 2 & 3). Osaka had the lowest number of diseased fruits at 10.38, Tanjung had the most at 12.02 infected fruits, and Unpad came in second at 11.75. With 18.39 fruits, the rain shelter had the highest disease number among the growing microclimates; the open field followed it at 15.03 fruits, and the screen house had the lowest incidence, with 2.67 fruits. When it comes to watering regimens, the 25% ETc level had the highest diseased fruits with 14 fruits, 50% ETc, and 75% ETc with 12.64 and 10.08 fruits, respectively, and the 100% ETc level with 9.86 impacted fruits, which had the lowest incidence (Fig. 10).

### Fruits affected by pest

**Fig. 11:**
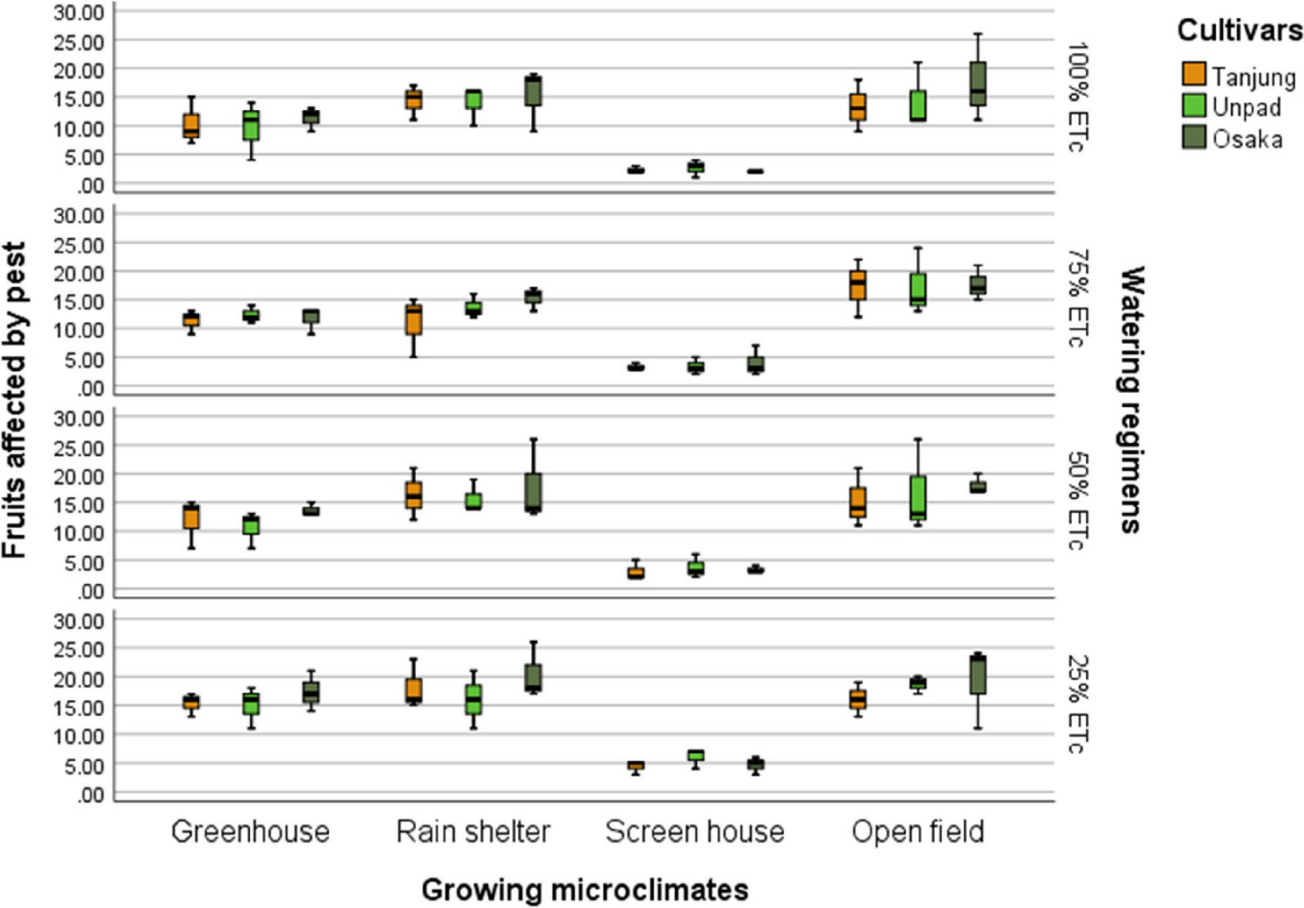
Mean fruits affected by pest of chili pepper cultivars as affected by different growing microclimates and watering regimens

The study showed that cultivars, growing microclimates, and irrigation regimens significantly (*P* < 0.05) affected the frequency of pest-affected chili fruits (Tables 2 & 3). With 13.08 damaged fruits, Osaka had the most significant (*P* < 0.05) pest prevalence among the cultivars; Unpad and Tanjung came in second and third, with 11.85 and 11.48 infected fruits, respectively. The open field had the highest number of pest-affected fruits (16.80), followed by the rain shelter (15.64 fruits) for growing microclimates. The screen house had the fewest pest-affected fruits (3.36), compared to 12.56 in the greenhouse. As for watering regimens, the maximum pest incidence was observed at the 25% ETc level, with 14.25 afflicted fruits; 50% ETc and 75% ETc showed 12.17 and 11.53 impacted fruits, respectively. With 10.61 impacted fruits, the 100% ETc level had the lowest pest occurrence (Fig. 11).

### Recovery rate %

The study showed that the recovery rate of chili plants was significantly (*P* < 0.05) influenced by cultivars, growing microclimates, and watering regimens (Tables 2 & 3). With a recovery rate of 69.32%, Osaka led the cultivars, Unpad came in second with 65.82%, and Tanjung came in third with 64.85%. The screen house grew microclimate with an 86.12% recovery rate, followed by the greenhouse at 70.43%. The rain shelter had the lowest recovery rate, at 51.23%, while the open field had a recovery rate of 53.58%.

**Fig. 12:**
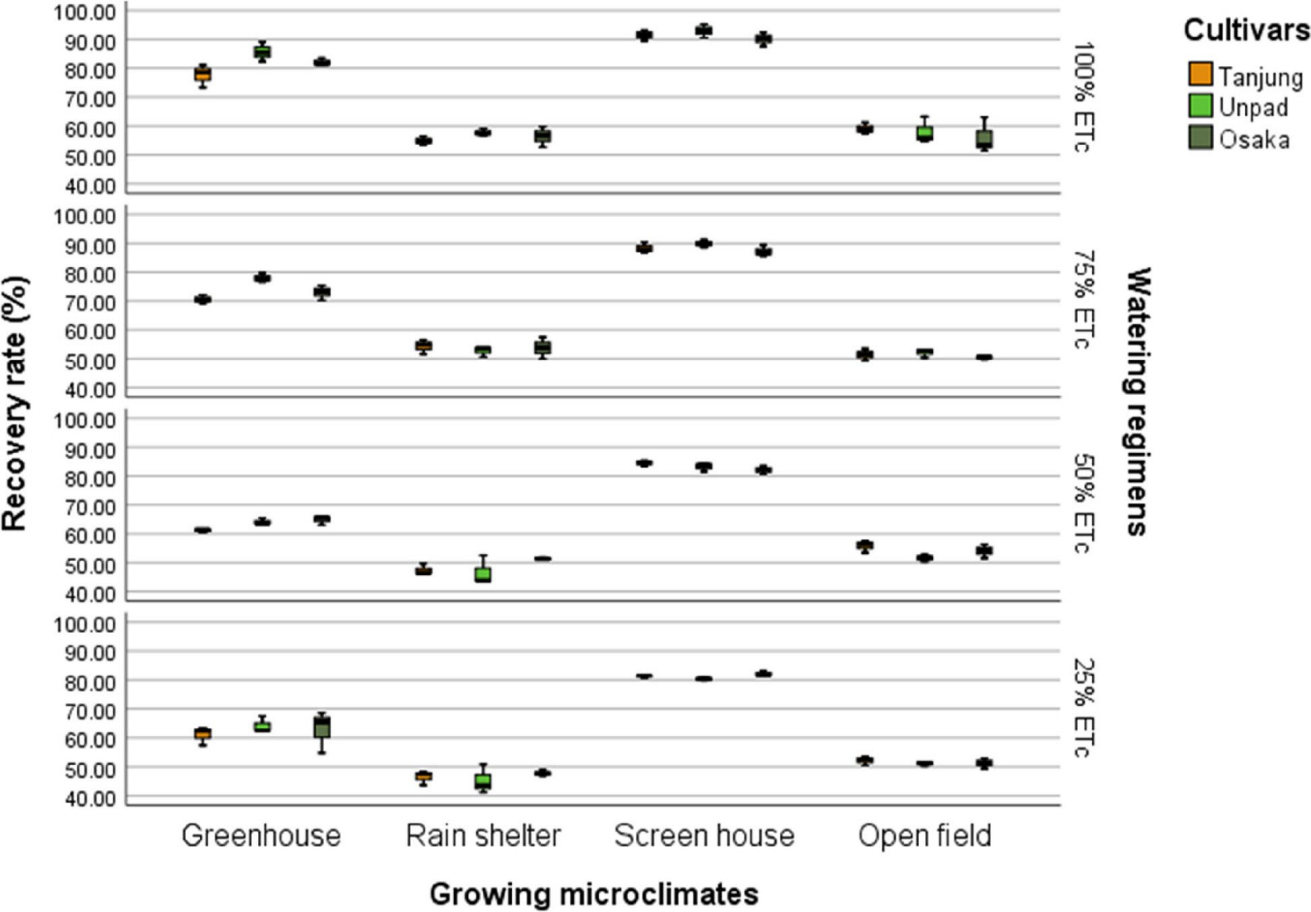
Mean recovery rate (%) of chili pepper cultivars as affected by different growing microclimates and watering regimens

In terms of watering regimens, the 100% ETc level recovered at the fastest rate (71.83%), followed by the 50% ETc level (62.25%) and the 75% ETc level (66.80%). With a recovery rate of 60.48%, the 25% ETc level had the lowest rate (Fig. 12).

### Correlation among observations

**Fig. 13:**
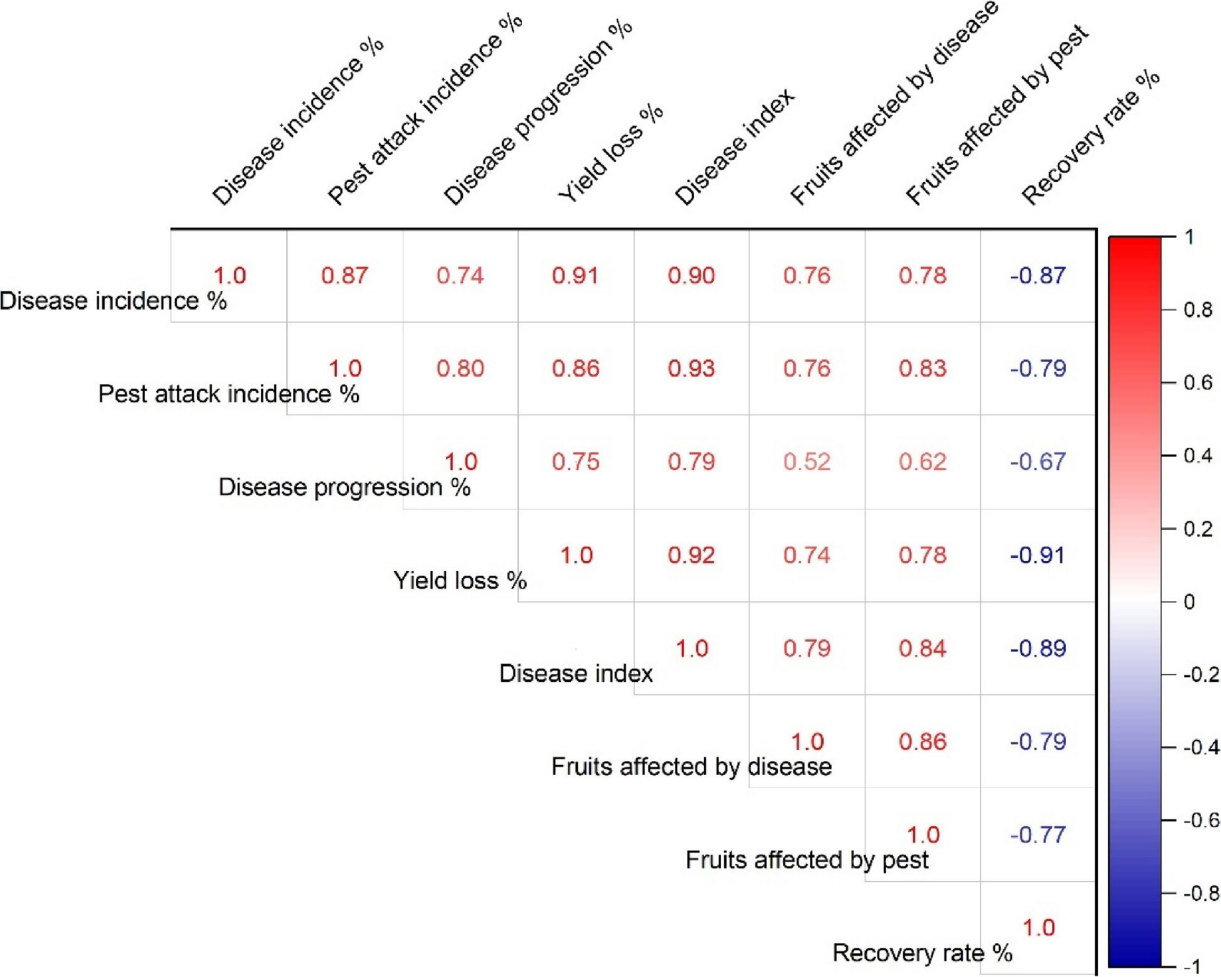
Pearson correlation of the studied observation in the experiment

The graph (Fig. 13) shows a correlation matrix that illustrates the correlations between several parameters linked to the cultivation of chilies, such as yield loss, disease index, fruit impacted by pests and disease, disease incidence, disease progression, and recovery rate. Positive correlations are shown in red, and negative correlations are shown in blue. The color intensity indicates the significance of the correlation. Incidence of pest attacks (0.87), disease index (0.90), and yield loss (0.91) all showed strong positive correlations. A strong positive correlation exists between yield loss and the occurrence of pest attacks (0.86) and disease index (0.92). Strong negative correlations have been observed between recovery rate and disease index (-0.89), yield loss (-0.91), and disease incidence (-0.87). Although the correlation is smaller than in other pairs, fruits affected by pests and disease positively correlate with other disease-related metrics.

### Disease nature and its severity

**Fig. 14:**
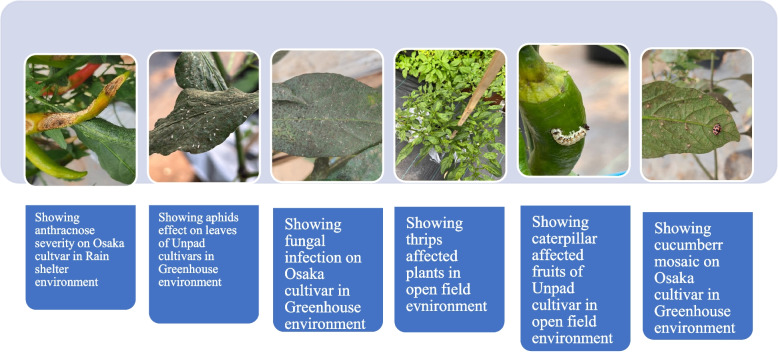
Showing major disease affected plants, fruit and leaves in the study areas

Considering the disease severity in cultivars (Tables 4 & 5), The Osaka cultivar had higher rates of disease severity for anthracnose (6), powdery mildew (4), cucumber mosaic (6), and pepper mottle (6) (Fig. 14). In contrast, the Unpad cultivar had higher rates of disease severity for bacterial wilt (4), fusarium wilt (5), and root rot (2). The greenhouse design was most significantly impacted among the various growing designs, with severity ratings of 6 for anthracnose, 6 for bacterial wilt, 5 for powdery mildew, and 7 for fusarium wilt. In contrast, anthracnose (6), fusarium wilt (7), cucumber mosaic (7), and pepper mottle (7) all exhibited greater severity rates in the rain shelter environment. The disease severity was shown to be most affected by watering regimens with a 25% irrigation volume. Higher disease severity rates were noted for root rot (5), bacterial wilt (6), fusarium wilt (7), cucumber mosaic (7), and pepper mottle (5).

**Table 4:**
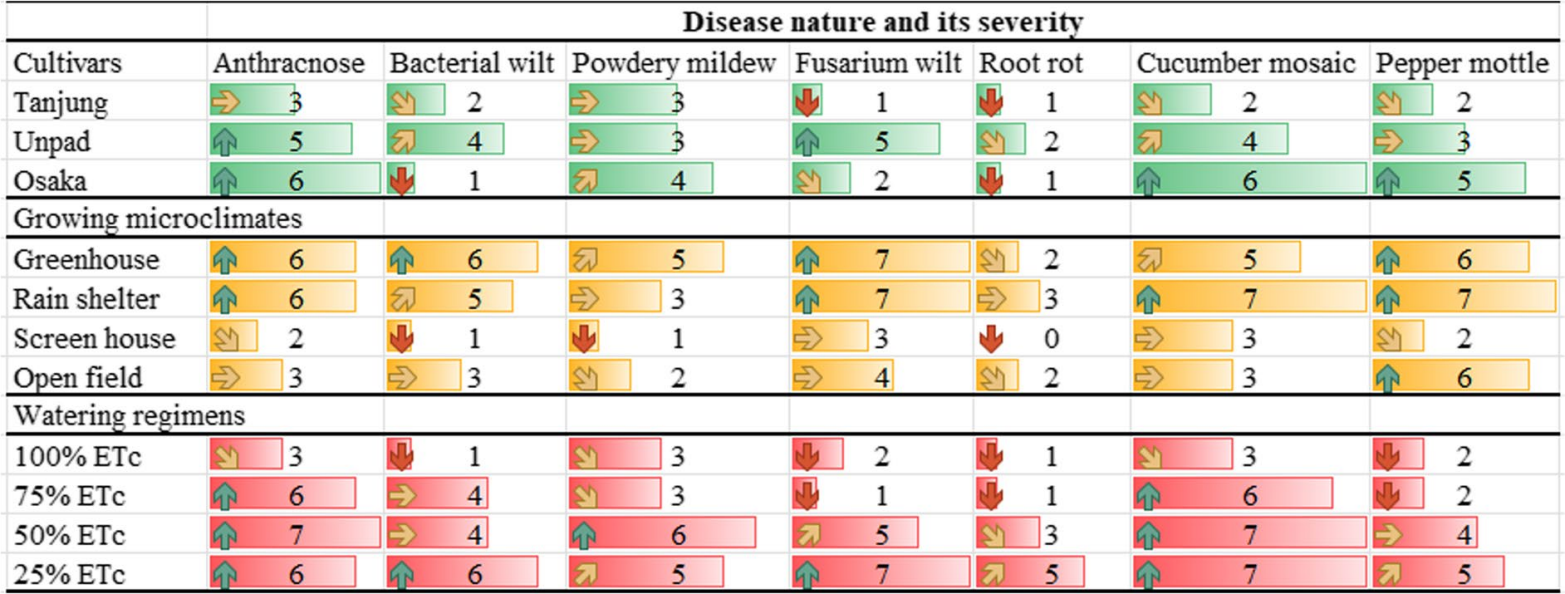
Major disease natures and its severity among the studied parameters

Severity rating and criteria for analysis (1-10 ratings) is mentioned in Table 1 (Methodology)

**Table 5:** Showing the disease nature and its major impact on fruits rotting

Disease	Nature of disease	Effect on fruit
*Anthracnose*	Fungal infection	Sunken lesions, dark spots, fruits rotting
*Bacterial Wilt*	Bacterial infection	Wilting, yellowing, necrosis, fruit rotting
*Powdery Mildew*	Fungal infection	White powdery patches on fruit surface
*Leaf Curl Virus*	Viral infection	Malformation, mottling, stunted fruits
*Fusarium Wilt*	Fungal infection	Yellowing, wilting, rotting
*Root Rot*	Fungal infection	Reduced growth, eventual rotting
*Cucumber Mosaic*	Viral infection	Mottling, distorted growth, reduced fruit size
*Pepper Mottle*	Viral infection	Mottling, reduced fruit size, distorted growth,
*Tomato Spotted*	Viral infection	Spots, distorted growth of fruit, mottling

### Pest nature and its severity

When it came to the severity of the pests (Tables 6 & 7), the Osaka cultivar got higher for white fly (3), thrips (6), leaf miners (7), and spider mites (6), while the Tanjung cultivar rated higher for caterpillars (6) and spider mites (6). Similar to this, out of all the emerging designs, the rain shelter design was most greatly impacted. Spider mites (6), leaf miners (7), and white flies (7), in comparison. In contrast, the severity of caterpillars (7) and white flies (7) was found to have a significant impact on open fields (Fig. 14). It was shown that irrigation volumes of 25% or more had the greatest impact on the intensity of the pests. Leaf miners (6), thrips (7), and white flies (5) all had higher pest severity rates discovered. A higher incidence of caterpillar and spider mite infestations (severity rate of 7) was also seen with 50% watering.

**Table 6:**
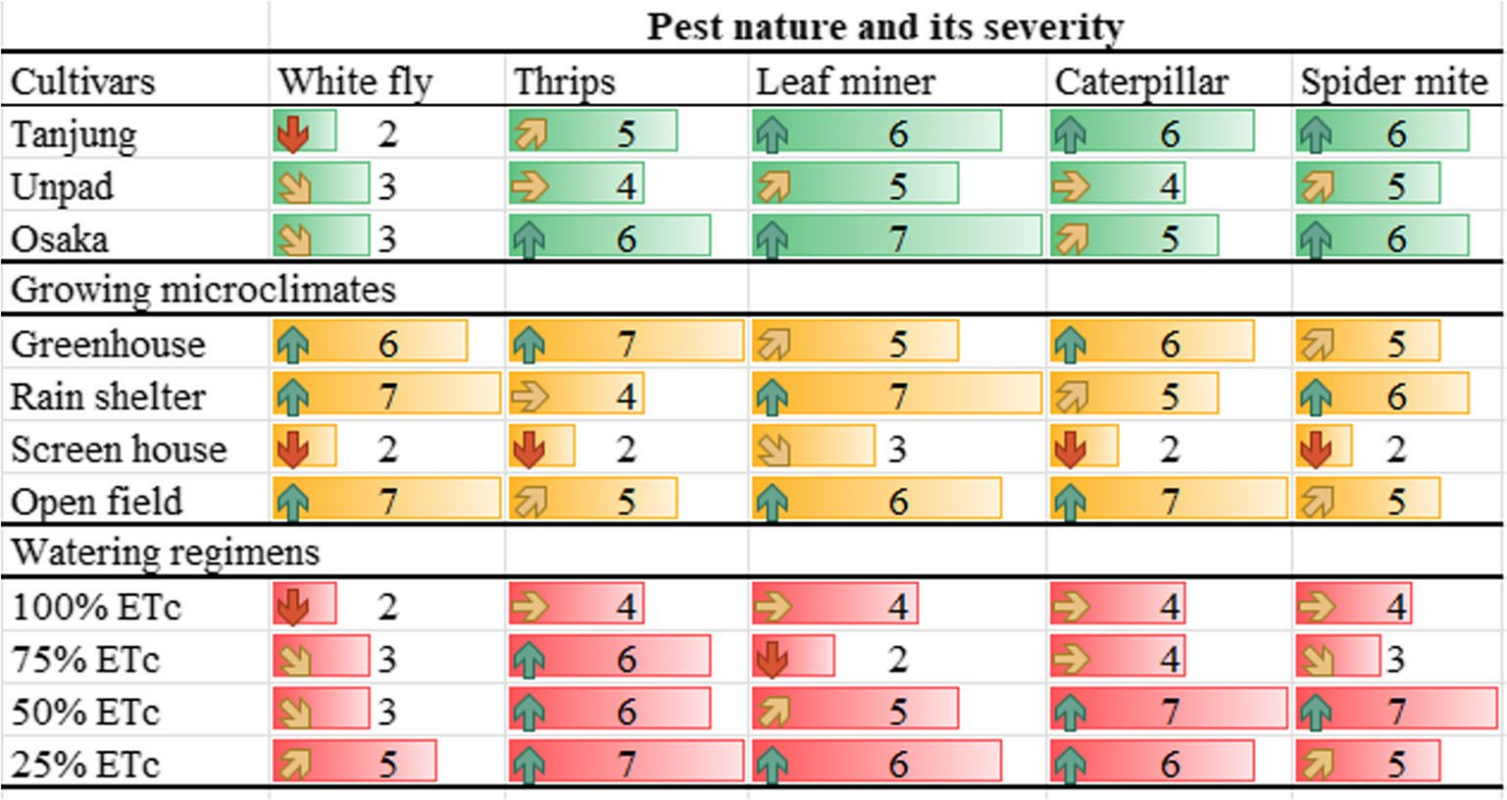
Major pest natures and its severity among the studied parameters

**Table 7:** Showing the pest nature and its major impact on fruits rotting

Pest	Nature of Pest	Effect on Fruit
*Aphids*	Sucking Insect	Curling, distortion, stunted growth, yellowing
*Whiteflies*	Sucking Insect	Yellowing, honeydew secretion
*Thrips*	Sucking Insect	Scarring, silvering, distortion
*Leaf Miners*	Leaf-Mining	Tunnelling, leaf discoloration
*Caterpillars*	Chewing Insect	fruit decay, scars, Holes, feeding damage
*Beetles*	Chewing Insect	fruit decay, Holes, scars, feeding damage
*Spider Mites*	Piercing/Sucking	Webbing, Yellowing, stippling, reduced fruit quality

## Discussion

Climate change can modify the growth stage, development pace, pathogenicity of infectious agents, resistance and physiology of the host plant due to variations in temperature and precipitation regimes [19]. Increased day to night temperature variations in open fields (Fig. 1) can cause stress to plants and increase their disease susceptibility [20]. Excessive light and heat can stress plants and generate microclimates that encourage the development of diseases on their leaf surfaces, even as adequate sunshine can support healthy plant growth [4, 21]. Screen houses provide great ventilation and drastically lower humidity (Fig. 2), which makes it harder for pathogens to thrive. By absorbing intense sunshine and allowing just the right amount of light, the screen creates an ideal growing environment that increases plant resistance to disease [10]. The increased availability of food and pest-friendly environmental factors, such as the prevalence of weeds and other plants that offer refuge and nutrition, maybe the cause of the rise in pest attacks in open fields [3]. The majority of pests are effectively kept out of screen houses by their physical barrier, which considerably reduces the frequency of pest infestations [17]. The increased exposure to pests and environmental stressors in the open field and rain shelter areas may be the cause of the higher yield loss [22]. The rain shelter might need more protection from pests; the open field might offer more conducive circumstances for pests to flourish. Although it's a controlled environment, the greenhouse may have a high disease index because of its poor ventilation and greater humidity (Fig. 2), which encourage the growth of pathogens [22, 23]. Variable weather, including temperature fluctuations and humidity changes, can make open fields more vulnerable to disease growth and erode plant defenses (Figs. 1, 2, 3, 4). Open fields provide an effortless atmosphere for disease transmission through wind, splashing rain, and insect vectors [15]. Different growing conditions may support diverse populations of pest competitors and natural predators [11, 24]. Because of its controlled environment, a screen house, for instance, can have more beneficial insects or microorganisms that feast on or compete with pests, thus lowering the number of pests [25]. Although the rain shelter offers some protection from rainfall, it may nevertheless provide an environment that is favourable to the propagation of disease, especially for diseases that prefer humid conditions [26]. The variations in pest severity throughout growth patterns emphasize how crucial it is to take environmental control methods and routine monitoring into account to lessen the effects of pests [27].

Inadequate water supply can hinder the uptake of nutrients, weakening the plants' defences against infections. Drought-stressed plants can release volatile substances that draw pests and make pest assaults more likely [18]. Regarding watering schedules, insufficient hydration may be the cause of the significant output losses at 25% and 50% ETc, which would affect plant growth and metabolic processes [27–29]. The prolonged leaf wetness caused by constant exposure to rain and dew encourages the growth and dissemination of bacterial and fungal infections [30]. The higher pest incidence in lower watering applications may make an environment more conducive to pests that frequently flourish in areas with little water availability [31]. Varying irrigation levels and growing environments can influence the plant's ability to protect itself against pests. Stress from either too much or too little water can weaken a plant's defences, leaving it more susceptible to pest attacks [32]. The decreased water supply may be the cause of the slower recovery rate, which could stress the plants more and make them more vulnerable to diseases and pests [33]. Due to the increased stress brought on by water scarcity, plants with limited water supply may recover more slowly. The plants are probably under stress from the reduced irrigation volume, which increases their susceptibility to diseases and hinders their capacity to mount a strong defence [34]. The physiological status of plants can be weakened by shallow watering, which exacerbates the severity of diseases [35]. Plants that receive less water than ideal, either too little or too much, may become stressed and more susceptible to insect infestations [36] (Fig. 13). Plants that receive too little water may have weakened defences against pests, and those that receive too much water may have created humid environments (Fig. 2) that are conducive to the growth of pests [37].

The premise that breeding for disease resistance can be temporary due to the changing nature of pathogens and pests is consistent with the non-significant effect of cultivars, indicating that the genetic makeup of the crop plants did not provide a significant defence against pests [38]. Tanjung may be less tolerant of environmental stresses such as changes in humidity, temperature, and light intensity, which could result in a more significant yield loss (Figs. 1, 2, 3, 4) [39]. The cultivar may be less resistant to diseases and pests common in the environment, which would increase production loss [11]. The elevated humidity and decreased ventilation in the rain shelter, which provides the perfect environment for the spread of disease, are probably the cause of the high number of affected fruits [10, 40] (Fig. 13). The genetic composition of the Osaka cultivar may provide greater resilience to diseases, accounting for the cultivar's decreased incidence of diseased fruits [41]. For instance, genetic selection or breeding initiatives have created specific cultivars with increased disease resistance. The rate of recovery may also be influenced by the cultivars' habits of growth [42]. Specific cultivars, for instance, can grow more compactly, which could make them more vulnerable to stress and slower to recover. These findings imply that cultivar-dependent vulnerability to specific diseases occurs [43]. The Osaka cultivar's increased vulnerability to fungi and viral diseases may be related to its genetic composition; molecular research could explore this further [16]. The Osaka cultivar's higher pest severity may be caused by physiological features that attract these pests, nutritional value, or leaf morphology [15].

## Conclusion

Despite minimal cultivar selection, this study shows that growing microclimates and watering regimens significantly impact disease incidence and pest attacks in chili farming. Screen houses demonstrated the preventive advantages of regulated environments, with the lowest rates of disease and insect incidents found in open fields. Higher watering levels (75% and 100% ETc) indicated lower impacts, while lower levels (25% and 50% ETc) linked with higher disease and pest prevalence. These results imply that reducing disease and insect problems in chili production requires careful attention to microclimate conditions and watering regimens. Farmers can increase the output of chilies, lower crop losses, and improve overall crop quality by using improved growing conditions and scheduling for watering. It can result in more productive and sustainable agricultural methods.

## Data Availability

All of the research data are presented in this manuscript.

## Data availability

All of the data regarding this manuscript have been presented here. There is no other data concerning to this paper.
